# Four-Dimensional Determination of the Patient-Specific Centre of Rotation for Total Temporomandibular Joint Replacements: Following the Groningen Principle

**DOI:** 10.3390/jpm12091439

**Published:** 2022-08-31

**Authors:** Bram B. J. Merema, Max J. H. Witjes, Nicolaas B. Van Bakelen, Joep Kraeima, Frederik K. L. Spijkervet

**Affiliations:** Department of Oral and Maxillofacial Surgery, University Medical Center Groningen, University of Groningen, Hanzeplein 1, P.O. Box 30.001, 9700 RB Groningen, The Netherlands

**Keywords:** mandible, jaw, 4D, motion analysis, kinematic, patient-specific, custom, prosthesis, TMJ, TJR, 3D-VSP, virtual surgical planning

## Abstract

For patients who suffer from severe dysfunction of the temporomandibular joint (TMJ), a total joint replacement (TJR) in the form of a prosthesis may be indicated. The position of the centre of rotation in TJRs is crucial for good postoperative oral function; however, it is not determined patient-specifically (PS) in any current TMJ-TJR. The aim of this current study was to develop a 4D-workflow to ascertain the PS mean axis of rotation, or fixed hinge, that mimics the patient’s specific physiological mouth opening. Twenty healthy adult patients were asked to volunteer for a 4D-scanning procedure. From these 4D-scanning recordings of mouth opening exercises, patient-specific centres of rotation and axes of rotation were determined using our JawAnalyser tool. The mean CR location was positioned 28 [mm] inferiorly and 5.5 [mm] posteriorly to the centre of condyle (CoC). The 95% confidence interval ranged from 22.9 to 33.7 [mm] inferior and 3.1 to 7.8 [mm] posterior to the CoC. This study succeeded in developing an accurate 4D-workflow to determine a PS mean axis of rotation that mimics the patient’s specific physiological mouth opening. Furthermore, a change in concept is necessary for all commercially available TMJ-TJR prostheses in order to comply with the PS CRs calculated by our study. In the meantime, it seems wise to stick to placing the CR 15 [mm] inferiorly to the CoC, or even beyond, towards 28 [mm] if the patient’s anatomy allows this.

## 1. Introduction

A total joint replacement (TJR) of the temporomandibular joint (TMJ) in the form of a prosthesis may be indicated for patients who suffer from severe TMJ dysfunction. Documented indications include end-stage degenerative joint disease, recurrent ankylosis, and congenital disorders affecting the TMJ when joint saving approaches do not suffice [1]. Other indications for TJRs are condylar loss as a result of trauma or neoplasia in or near the joint or to replace a failed alloplastic or autogenous reconstruction [2]. In most of these patients, mandibular movement is impaired due to either anatomical changes or surgically caused scarification, often resulting in pain, difficulties in speech, impaired oral function, and limited maximum mouth opening.

When replacing the TMJ with a TMJ-TJR prosthesis, the condyle or its remnants together with the articular disc are removed in order to fit the prosthesis. This results in the removal of the insertion of the main muscle responsible for anterior movement of the condyle, the lateral pterygoid muscle, from its insertions at the mandibular condyle and articular disc. Removal of this muscle’s insertion site in order to place a TMJ-TJR is reported to decrease the amount of anterior movement of the TMJ from approximately 16 [mm] to only 2 [mm] [3] or less, thereby reducing the joint’s movements to near mere rotations [4]. The consequences of placing a TMJ-TJR unilaterally are a lack of anterior movement leading to asymmetrical mouth opening movements, where the mandible deviates towards the affected side, marginal laterotrusion towards the unaffected side [5], and unnatural loading of the contralateral joint [6].

**Figure 1:**
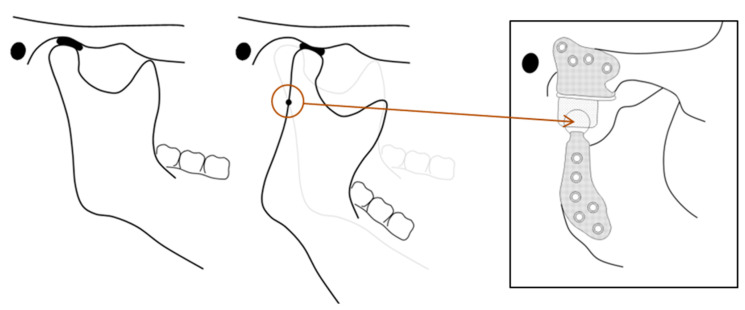
This sketch shows the effect of considering a fixed centre of rotation which is positioned inferior to the centre of the condyle. This lowered centre of rotation mimics the natural translational movement of the condyle whilst merely rotating. The left sketch shows the occlusal mandibular position. Middle shows both the occlusal and maximum opened position of the mandible which is obtained by pure rotation around the dot. The right picture shows the implementation of this effect in the Groningen TMJ-TJR prosthesis, according to the *Groningen Principle*.

To overcome this effect, the Groningen TMJ-TJR prosthesis was developed [7,8,9]. Apart from its unique feature that allows for free translational movement of the neo-disc, the prosthesis applies a lowered centre of rotation (CR) in relation to the anatomical condylar centre [8]. Prior research suggested that a lowering of 15 [mm] in relation to the condyle would be optimal as a fixed CR for unilateral TMJ prostheses [3]. This study was, however, based on 2D optical movement tracking with no direct relation to the bony anatomy and thus the condyles of the mandible [10]. The Groningen TMJ-TJR prosthesis has been available as a patient-specific (PS) device since 2017, opening doors to also personalise the position of the CR [8]. Figure 1 illustrates the effect of a lowered centre of rotation with the Groningen TMJ-TJR prosthesis.

Physiological mandibular movement is complex and, as per definition, not truly translatable to a mere rotation around a single axis. However, as mentioned, the movement of a TMJ reconstructed by means of a TMJ-TJR prosthesis should predominantly show rotational movement [4]. Since any translational movement cannot be expected to occur in the reconstructed TMJ due to a lack in lateral pterygoid muscle function, we chose to analyse a fixed centre of rotation, even though the Groningen TMJ-TJR allows for some free translation of the neo-disc [8].

When considering a fixed CR of the mandible, placing it more inferiorly should result in increased anterior movement of the associated condyle during mouth opening. Moreover, shifting the CR in a posterior direction should enable relatively more rotation in the coronal plane and, thus, in a more inferior excursion of the condyle [3] (Figure 1).

Several prior authors have succeeded in analysing the movement of subjects’ mandibles by means of tracking them in 2D (sagittal) or 3D [11,12,13,14,15,16,17,18]. Generally, incisal and condylar points are traced during mandibular movement to analyse the mandible’s paths of movement, whilst in other cases the mouth opening or closing are described by a path of changing instantaneous centres of rotation throughout the movement. The obtained movement tracking data, or four-dimensional (4D) data, of the mandible could also be used to calculate the CR in a PS manner. This, however, means the patient has to have a physiologically correct movement pattern of the mandible in order to determine these points correctly. Patients who are in need of a TJR of the TMJ often have an affected mouth opening. In such cases, a patient-specific (PS) determined CR of the prosthesis cannot be derived from mandibular movement exercises and so should be determined by alternative means.

The aim of this current study was to develop a 4D-workflow to ascertain the PS mean axis of rotation, or fixed hinge, that mimics the patient’s specific physiological mouth opening. The aim was to use this 4D-workflow to find out if the aforementioned prior determined 15 [mm] lowering in CR^3^ is still relevant and, if not, to suggest a PS CR location.

## 2. Materials and Methods

Twenty healthy adult patients who required cone beam computed tomography (CBCT) scanning for 3D virtual surgical planning (VSP) of their bilateral sagittal split osteotomy (BSSO) procedure between January 2020 and December 2021, were asked to volunteer for a 4D-scanning procedure. The inclusion requirements for the 4D-study were the presence of orthodontic brackets and the absence of TMJ dysfunction. Furthermore, the patient should be able to freely move the mandible without pain and other limiting factors. Before commencing with this study’s protocol, approval was received from the Medical Ethical Board, file number: METc 2020/355.

**Figure 2:**
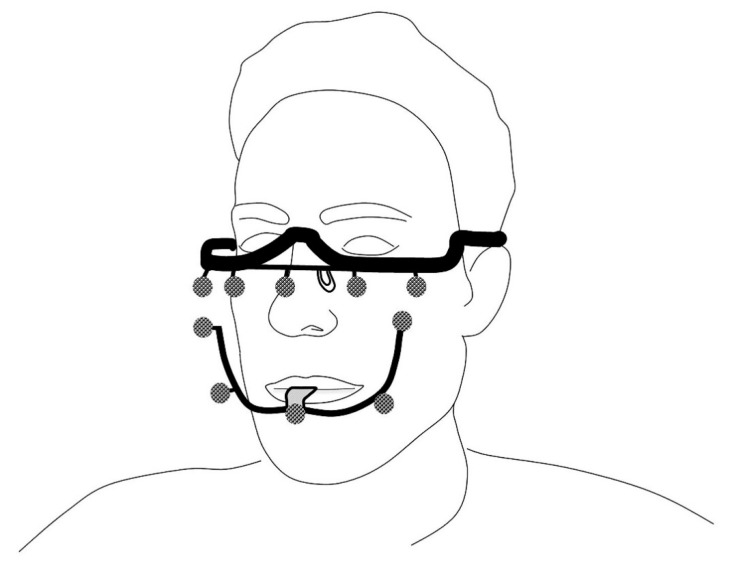
Sketch showing the 4D-CBCT setup. The subject is scanned with CBCT and subsequent 4D optical scanning in one procedure. The subject wears a maxillary frame resting on the nasal bridge and ears and a mandibular frame which is rigidly connected to the dental elements. Both frames are provided with five reflective markers that are visible on both the CBCT imaging and 4D optical tracking.

The 4D-scanning was performed with a 4D optical tracking module (Planmeca 4D Jaw Motion) installed in a CBCT scanner (Planmeca ProMax, Planmeca, Helsinki, Finland). The resolution of the CBCT images was 0.4 × 0.4 × 0.4 [mm] with a field of view of 230 [mm]. The subjects had to wear a polyamide maxillary frame, which rests on the nasal bridge and ears, and an aluminium mandibular frame rigidly connected to the lower dental arch and orthodontic brackets by means of an easily removable dental bite registration putty (Exabite TT NDS, GC America INC. Alsip, IL, USA). Both the maxillary frame and the mandibular frame accommodated five optical tracer spheres which could be optically recorded by the system (Figure 2). The CBCT scan was performed in maximum dental occlusion with the patient sitting up straight and in natural head position. The field of view was set to include the complete mandible and the maxilla as well as the orbits. Subsequently, movement exercises were carried out and recorded in real time with a frame rate of 24 [Hz]. 

The recorded experiments comprised five consecutive voluntary maximum mouth opening exercises per patient. The recorded data were exported as transformation matrices describing the transformation from the CBCT image to the mandible position in each frame. The transformation matrices were saved as .xml files and subsequently converted to .xslx format using Excel 2019 (Microsoft, Redmond, Washington, USA) to allow for easier access of the data for further analysis.

**Figure 3:**
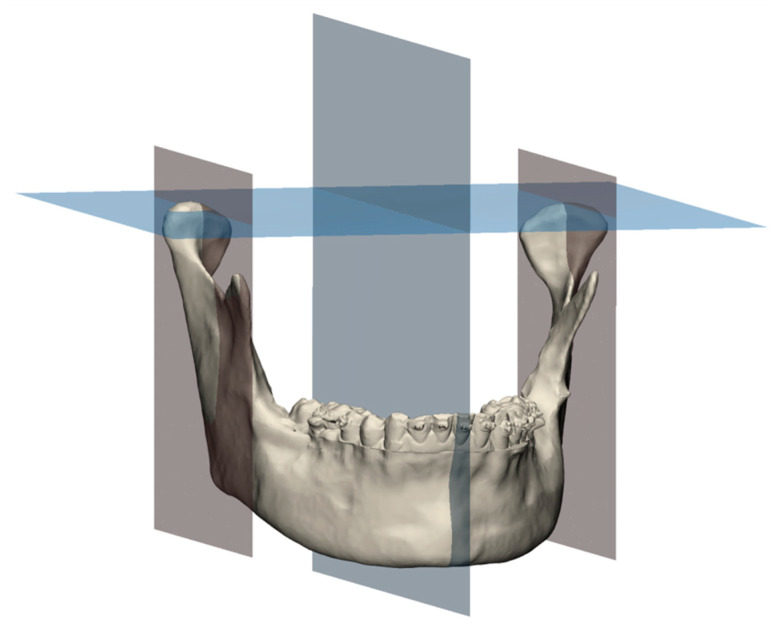
An example of the assigned Frankfurt horizontal plane and matching midsagittal plane (blue). The midsagittal plane was used to create two parallel mid-condylar planes (red), which intersected the condyles in their medio-lateral middle point. These mid-condylar planes were used in the JawAnalyser tool to calculate the patient-specific centres of rotation and, thereby, the patient-specific axis of rotation for the mouth opening.

The CBCT scan segmentations were performed in the Mimics 22.0 software (Materialise, Leuven, Belgium) and 3D-models of the mandible and maxilla were created. These models were imported into the 3-Matic Medical 15.0 software (Materialise, Leuven, Belgium) to determine the Frankfurt horizontal plane (FHP) and orthogonally positioned midsagittal plane. Parallel to the midsagittal plane, planes were created in the medio-lateral middle of each condyle. These mid-condylar planes were 100 × 100 [mm] in dimension and triangulated with a maximum edge length of 0.2 [mm]. Subsequently, the mid-condylar planes were merged with the 3D-model of the mandible and exported together with the maxilla model as standard tessellation language (STL) files (Figure 3).

A program was written to develop the JawAnalyser tool in MATLAB R2020a (Mathworks, Natick, Massachusetts, USA) to analyse the recorded 4D-data and to find the instantaneous centre axis of rotation of the moving mandible. The STL models of the maxilla and mandible were imported into this tool together with the subjects’ mid-condylar planes and 4D transformation matrix recordings. Start and end frames were chosen manually for each mouth opening, where the start frame was the maximal occlusal position of the mandible prior to the specific mouth opening and the end frame was the maximal open mouth position. The JawAnalyser compares the orientation of the planes of the mandibles’ start frame with the opened mandible’s planes and finds the point of least translation, and thus maximum rotation, on each plane. These points describe the start and endpoint of the mandible’s rotation axis or instantaneous centre of rotation. This 2D technique relies on the Reuleaux method [19] and is translated to 3D by applying two planes to the mandible to find the instantaneous centre of rotation. 

The accuracy of both the JawAnalyser tool and the entire workflow was validated. The JawAnalyser tool was validated by means of inputting geometries that were manually translated and rotated in space by known quantities and the results of the tool were compared to the known input translation and rotation values. 

**Figure 4:**
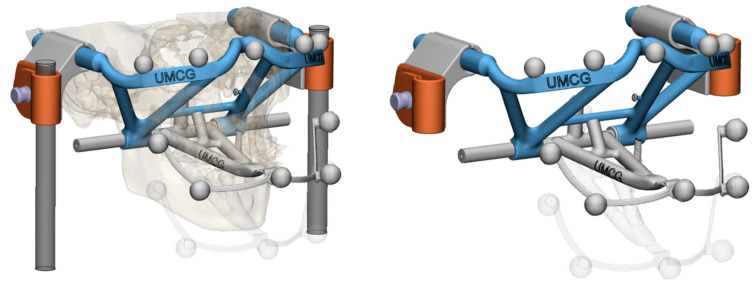
The phantom model that was designed in-house (University Medical Center Groningen /UMCG) for validation of the 4D-workflow. From a subject’s scan (**left**), the positions of the optical tracers were determined and the maxillary and mandibular frames were adapted to form a scaffold that could be rigidly connected to the CBCT scanner. The phantom allowed for rotation along the mandibular axis (**right**), resulting in a simulated 25 degrees opened mouth (transparent).

A phantom model was designed for validation purposes (Figure 4). The entire workflow, starting from the 3D-printing of the phantom, followed by the CBCT imaging, the subsequent 4D-recordings, the segmentations, and the final determined rotation axis, was validated with the aid of this 3D-printed phantom model. This model was based on the dimensions of a human head wearing the maxillary and mandibular tracer set-up, depicted in Figure 2, and was fixed to the CBCT scanner’s head-supports. It included the same optical tracer sphere positions as those of our subjects (Figure 2 and Figure 4). The mandible part of the phantom was then rotated 25 degrees around a fixed axis, with ten repetitions, comparable to a mouth opening of approximately 38 [mm]. The rotation axes were then determined using JawAnalyser and compared to the known physical positions of the phantom’s rotation axis. The begin and end locations of the rotation axes were determined in the mid-condylar planes and the left and right coordinate sets were registered.

Five pairs of coordinates, indicating the extremities of the axis of rotation for a specific mouth opening, were extracted for each subject from the JawAnalyser tool. The matching start and end frames of these five mouth openings were exported from the JawAnalyser tool as STL files and imported together with the extracted axes of rotation into 3-Matic. Then, all the start frames were matched to the CBCT occlusion mandible position whilst the matching axis of rotation was moved along with its mandible. This was necessary to normalise all the axes positions due to slight changes in occlusal positions. Once brought to the CBCT position, all the rotation axis coordinates were finalised and imported into Excel 2019 to calculate the mean x-, y-, and z-coordinates and one mean axis of rotation per subject.

**Figure 5:**
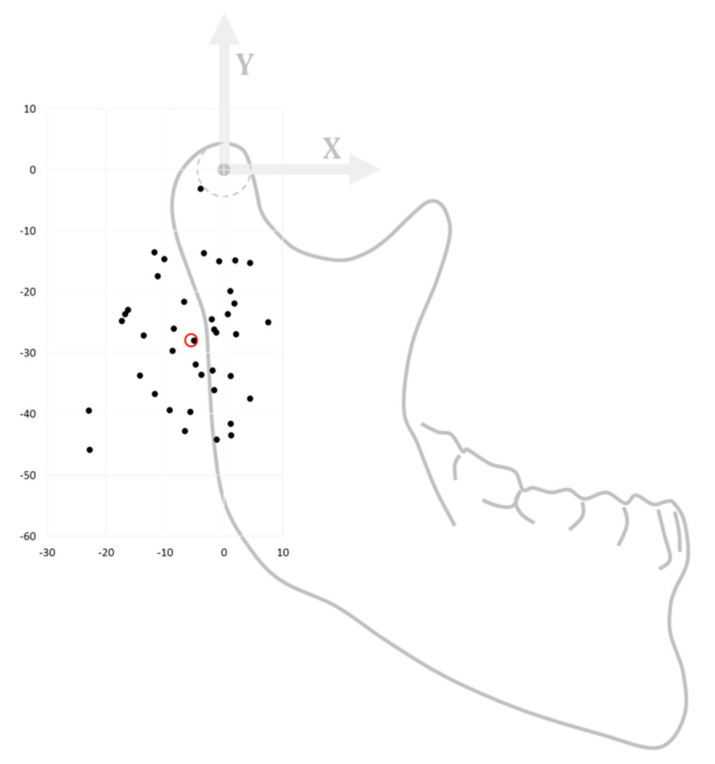
A scatter plot showing the 40 patient-specific centres of rotation coordinates we determined for our cohort (left & right sides). They are overlaid onto a generic mandible for reference, where the (0, 0) point lies in the centre of condyle point (CoC). The red circle indicates the mean measurement, (−5.7, −28.3).

Using the previously determined mid-condylar planes, a circle was sketched on the cross-section of the condyle. This circle matches the top radius of each condyle and its centre, the centre of condyle (CoC), defines the subject’s zero-point, which we used to quantify the position of the patient-specific mean axis of rotation (Figure 5).

The PS CR calculation was carried out for both lateralities in all subjects, resulting in 40 measurements. Regarding each CR, both a Δ x- and Δ y-distance from the CoC were registered in [mm]. The positive x-axis was placed along the FHP in an anterior direction, whilst the positive y-axis was positioned orthogonally to the FHP in a cranial direction. The measured CRs per subject (left and right) were considered independently of each other due to the amount of asymmetry. The data analysis was carried out in in IBM SPSS statistics version 23 (IBM corp., Armonk, NY, USA).

The manual determination of the CoC location was repeated by a second observer for ten condyles (BM and JK). The inter-observer variability calculation was carried out in the SPSS software. The inter-observer variability was supported by calculating the interclass correlation coefficient (ICC), whereby a value of <0.40 is poor, 0.40–0.59 fair, 0.60–0.74 good, and 0.75–1.00 is excellent [20]. This statistical test is an indicator for the reproducibility of our CoC location determination between different observers.

In order to visualise the effect of the calculated mean CR for each patient, points were placed at the inter-incisal point and both CoCs. These points were moved along with the mandible’s opening movements so that their coordinates formed paths. These paths, or traces, were then compared to four scenarios. The first was the physiological opening movement we measured with our 4D-tracking system. The second was a simulated opening movement with a pure rotation around the PS calculated mean CR. The third and fourth scenarios were simulations of the left condyle following its physiological path while the right joint was replaced by a fixed CR, either 15 [mm] inferiorly to the CoC or at the calculated PS mean position.

## 3. Results

Twenty healthy adult subjects were included in this study. These volunteers were 12 males and 8 females, aged from 18 to 53 years, with a mean age of 29. All the subjects had complete natural dentition, wore orthodontic brackets in at least the lower front region, and had no temporomandibular joint dysfunction. 

Validation of the JawAnalyser tool resulted in a perfect match between the calculated and input CRs regarding the geometries that were manually rotated and combined, as well as rotated and translated, as is the case in mandibular kinematics. To validate the entire workflow, including the CBCT imaging, the 4D recordings, the segmentations and the final traces, the rotation axes of all ten mouth opening movements were determined for the phantom model. The mean Euclidean error of the start and end coordinates of the rotation axes to the true coordinates of the phantom was 0.81 [mm]. 

The 40 CR measurements were normally distributed so the mean position was considered to be a relevant indicator. The mean CR of the right-sided joints was located 28.3 [mm] inferiorly and 5.7 [mm] posteriorly, whilst the mean CR of the left joints was located 27.6 [mm] inferiorly and 5.2 [mm] posteriorly to the CoC. When both literalities were combined, the mean CR location was positioned 28 [mm] inferiorly and 5.5 [mm] posteriorly to the CoC. The ranges were (−45.9/−3.2 mm) and (−22.9/+7.5 mm) for the superoinferior and anteroposterior directions, respectively. The 95% confidence interval of the calculated mean Δ y-distance from the CoC was −22.9 to −33.7 [mm] and −3.1 to −7.8 [mm] for the Δ x-distance. Figure 5 shows a scatter plot of the determined coordinates, indicating the PS positions of the CR, overlaid onto a mandible for reference. Table 1 depicts all the calculated CR positions per subject.

The manual selection of the CoC for both condyles in ten condyles was repeated by a second observer (BM and JK). The inter-observer variation was 1.47 [mm] with an interclass correlation coefficient (two-way mixed) of 0.997, indicating an excellent match for the measurements by both observers.

**Figure 6:**
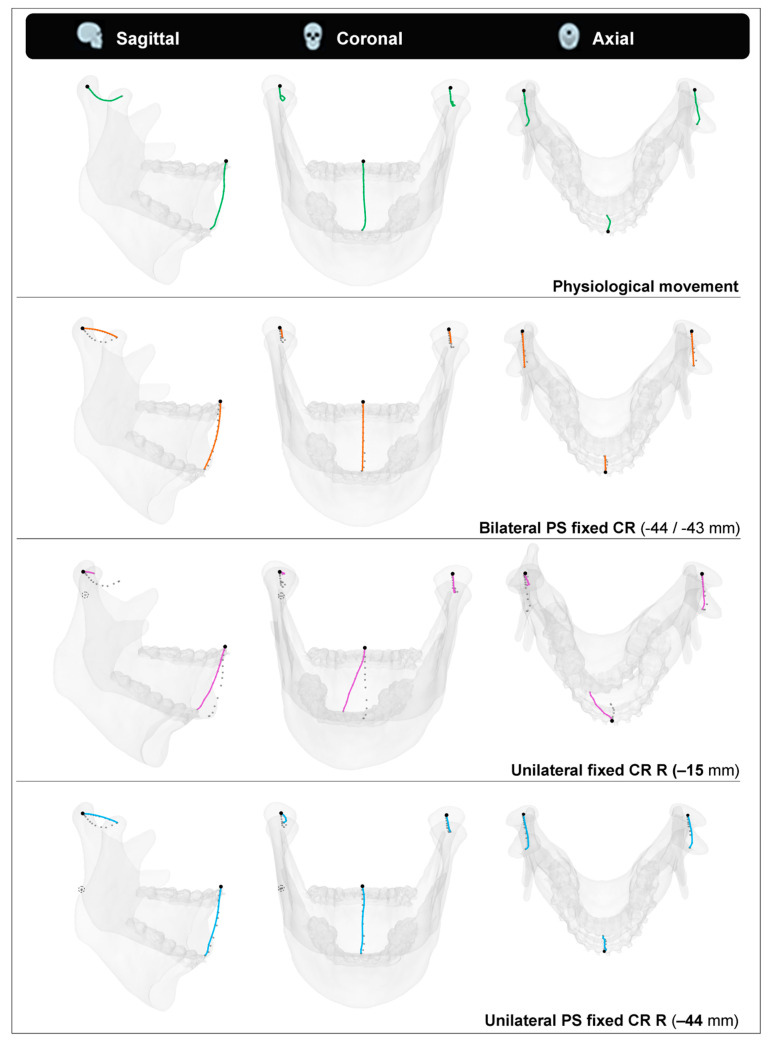
Condylar and incisal point traces describing the mouth opening movement in four different scenarios. Top to bottom: Physiological movement describes the actual recorded movement pattern for this specific subject. Bilateral PS fixed CR describes a simulated mouth opening around the patient-specific (PS) axis of rotation determined with our workflow. Unilateral fixed CR R describes a simulated mouth opening with a fixed centre of rotation (CR) at 15 mm inferior to the centre of condyle (CoC) and the physiological movement of the left condyle. Unilateral PS fixed CR R describes a simulated mouth opening with a fixed CR at the PS determined position, 44 mm inferior to the CoC and the physiological movement of the left condyle. The dotted traces in the three simulated mouth openings indicate the physiological movement traces. Note the severe lateral deviation that occurs when the CR is positioned much closer to the CoC than PS determined.

Patient 19’s coordinate tracing throughout the mandibular opening during the four described scenarios is visualised in Figure 6. We chose this patient because of their rather inferior positioned CRs, which pronounces the differences between the four scenarios well. 

## 4. Discussion

The workflow and associated JawAnalyser tool developed in this study serve the purpose of determining the optimal PS fixed axis of rotation. The primary application of such PS rotation axes is in designing PS TMJ-TJRs. None of the commercially available TMJ-TJRs make use of a PS calculated CR in their designs, and their CR positions are based on, e.g., the anatomical condylar position / CoC [21] or on technical limitations, i.e., required minimal thicknesses of the used materials [22]. In the Groningen TMJ-TJR, however, the CR is placed 15 [mm] inferior to the CoC with the aim of mimicking the physiological movement [7]. In this prosthesis, the 15 [mm] CR can be easily substituted by a PS determined value due to its patient-specific design [8,9]. This is naturally within certain boundaries set by the surgical approaches used for implantation. 

The PS CRs determined in this study mimic the complex physiological mandibular mouth opening movement, which consists of both translational and rotational movements, by approaching the mouth opening as merely a rotational movement around the PS calculated rotation axis.

By taking all the patients in our cohort into account, we determined the mean position of the CR as being 28 [mm] inferiorly and 5.5 [mm] posteriorly to the CoC. The 95% coincidence intervals for the mean indicate the probability of the majority of the measurements lie within 23 and 34 [mm] inferiorly and 3 to 8 [mm] posteriorly to the CoC. Many prior researchers have studied the kinematics of the mandible [12,13,14,15,16,17,18], but the only study which determined a mean CR position that mimicked the physiological mouth opening was the one by van Loon et al. [3]. As mentioned before, they determined an optimal CR of 15 mm inferior to the CoC. Although they did not report specific CRs per subject, they did mention that for the determination of their optimal CR, the physical boundary conditions, i.e., the prosthesis dimensions, were considered as well. This influenced the determination of their optimal centre of location, and therefore, it was not the merely anatomical optimal centre of rotation. Lowering the CR by 15 [mm] with respect to the CoC can already be challenging in some of the smaller patients. Therefore, it does not appear feasible to directly implement the mean of the PS determined CRs to the Groningen TMJ-TJR or in any commercially available prosthesis in most of our entire set of patients. Lindauer et al. also observed a great variation in rotation axes during mouth opening, and they discussed the value of PS determination of CR [23]. 

After replacing the TMJ with a TJR-prosthesis, it can be assumed that the reconstructed joint will show, postoperatively, rotational movement only [4]. When substituting the physiological movement of the mandible with a mere rotation around the corresponding PS axis of rotation that was calculated with our JawAnalyser, we observed that the inter-incisal point closely matched the physiological trace in all the planes. Although the CoCs matched both the start and end positions, they had an inversely shaped trace when compared to the physiological trace due to the strict rotational movement. When we replaced the right TMJ with a fixed CR located 15 [mm] inferiorly to the CoC, thereby mimicking the replacement of this joint with a Groningen-TMJ-TJR prosthesis, the simulated mouth opening in this particular patient demonstrated an obvious deviation in both the sagittal and coronal planes. The lateral deviation of the inter-incisal point at maximum mouth opening (MMO), however, was more than 12 [mm] in this scenario. The excursion that would have been made by the right condyle if had it not been replaced by the TJR would have been only 6.4 [mm] as opposed to 21.4 [mm] in the measured physiological trace. This indicates that the PS CR for this patient’s joint was situated even further inferiorly than 15 [mm]. The latter scenario, where we maintained the left physiological condylar trace and substituted the right joint with a fixed CR at the PS calculated point, showed a perfect match between both the occlusal and MMO positions of the physiological mandible, and the lateral deviation at MMO was non-existent. 

The visualised effects of lateral and posterior deviations, as seen in the scenario with unilateral 15 [mm] lowering of a fixed CR (Figure 6), are less pronounced in patients with a smaller mismatch between the applied fixed CR position and the calculated PS CR position. It should be noted that these deviations are even more pronounced in TJRs with a CR that is positioned higher than 15 [mm], i.e., the Groningen principle. This applies to all commercially available TMJ-TJR prostheses. 

This leaves us with three options:

- accept asymmetrical mouth openings and closing movements
- alter the current prosthesis kinematic principles
- adapt the movements of the contralateral joint

Since the latter, entailing operating on and restricting a healthy joint, would be considered unethical, this means only changing the prosthesis concept or accepting a suboptimal mandibular movement. Even though conforming to the PS axis of rotation might not be physically feasible for all cases, knowing the patient’s specific axis of rotation is always valuable for predicting the outcome of the TJR procedure and to prepare the patient for the expected outcome.

**Figure 7:**
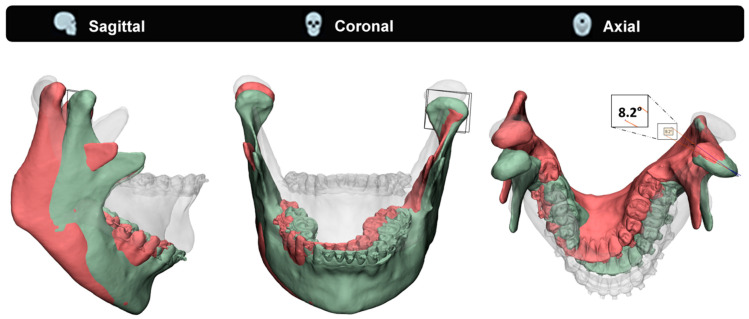
An illustration showing the effect of a wrongly positioned unilateral fixed centre of rotation (CR) simulating a TMJ-TJR prosthesis on the right side, on the healthy contralateral joint. In transparent the occlusal mandibular position is shown. Green indicates the maximum opened mouth according to the recorded physiological data, and red shows the simulated mouth opening according to scenario ‘Unilateral fixed CR R (−15 mm)’ in Figure 6. The unilateral fixed CR R (−15 mm) scenario is where the left condyle follows the physiological path, whilst the right side has a fixed CR applied too close to the centre of condyle position for this specific patient. Note again the severe lateral deviation of the red mandible and the unnatural rotation this forces on the contralateral healthy joint. In this specific case, the left condyle is forced to rotate an 8.2 degree angle in the axial plane upon opening compared to the physiological opened condyle position.

To illustrate the effect of a mismatched fixed CR on the contralateral healthy joint, we used an exemplary case to compare two condylar position scenarios (Figure 7). The measured physiological MMO position was compared to a simulated MMO in a case of a unilaterally fixed CR (15 [mm] inferior to the CoC), which is comparable with a unilateral TJR. In this particular case, the calculated PS CR is approximately 44 [mm] inferior to the CoC. The contralateral healthy joint completes its full translation, whilst the replaced joint only does approximately a third. As a result of the mismatch between the PS CR and the simulated CR (44 [mm] vs 15 [mm]), the mandible rotates in the axial plane, resulting in a contralateral healthy joint with a condylar seating that is forced to rotate at an 8.2 degree angle. Figure 7 illustrates this rotational error. The patient’s measured physiological maximum laterotrusive excursion, throughout the mouth opening, results only in a 1.5-degree angle, which is only 18% of the simulated forced rotation. The effect a forced rotation and change in condylar seating has on the healthy joint, as well as the maximum acceptable forced rotation angles, is still unknown. Additionally, the fact that commercially available prostheses have an even higher CR compared to the 15 [mm] inferiorly placed CR in our case means that, according to the Groningen principle, this effect would have been even greater in those TMJ-TJR prostheses [5,14].

In patients with restricted mandibular movement, e.g., due to severe unilateral ankylosis, performing a 4D-analysis can be challenging, if not impossible, depending on the severity of the restriction of movement. Furthermore, our proposed workflow would be inapplicable for clinicians who do not have access to 4D techniques. Regarding both situations, it could be worth exploring if the PS CR is related to the morphology of the mandible and fossa and can thus be predicted instead of measured. Among our cohort, we observed that a large portion of the calculated PS CRs lay on or close to the occlusal plane. This observation is supported by prior researchers’ findings [24].

In future work, we would like to test the hypothesis that PS CRs can be predicted based on the morphology of the mandible and fossa. Tools that can be applied to test such typical hypotheses are statistical shape modelling (SSM) [25,26] and principal polynomial shape analysis (PPSA) [27]. By using the segmentations of the mandible and fossa together with the calculated PS CRs from our cohort as input for the model, we can make the model predict the PS CRs of mandibles input without 4D data. Further validation of the SSM/PPSA CRs should indicate if there is any relationship between the mandible’s morphology and the position of its CRs. However, in order to establish a robust model, we would need to expand our current cohort.

The main limitation of this current study, apart from the relatively small sample size of 20 patients, is the homogeneity of our cohort. Being patients who required CBCT scanning for 3D VSP of their BSSO procedure, all patients in this study had a class II or III occlusion (17 vs. 3). Whether these types of malocclusions have an effect on the movement pattern of the mandible, especially mouth opening, remains unclear, as to the best of our knowledge, this cannot be found in the current literature. Scanning a cohort of control subjects might provide us with these answers, but ethics prevent us from CBCT scanning of healthy subjects.

The strengths, on the other hand, are the fact that our well-validated method turned out a feasible workflow that is not reserved for just BSSO patients. It addresses an issue that seems generally accepted or overlooked and to which more attention should be paid.

## 5. Conclusions

This study succeeded in developing an accurate 4D-workflow to determine a PS mean axis of rotation that mimics the patient’s specific physiological mouth opening. Our results strengthen the conception that PS determination of the CR as, e.g., used in TMJ-TJR prostheses, adds value in regard to mimicking the physiological mouth opening movement. The CRs applied in the commercially available TMJ-TJR prostheses are likely positioned too cranially for the bulk of the population, causing physiologically incorrect mandibular movements. The current PS Groningen TMJ-TJR prosthesis applies a lowered CR of 15 [mm] with respect to the CoC and thereby approaches the physiological movement of the mandible to some extent. The mean optimal CR we determined in this study, 28 [mm] inferior to the CoC, however, implies that 15 [mm] of CR lowering is not sufficient for the bulk of the population. In the Groningen TMJ-TJR and perhaps other commercially available prostheses, the amount of CR can easily be lowered to a PS determined CR within certain boundaries; however, due to technical and surgical constraints this would not be far enough to comply with the PS CRs of the majority of the patients.

Therefore, a change in TMJ-TJR prosthesis concept is necessary for all commercially available prostheses in order to comply with all PS CRs calculated in our study. In the meantime, it seems wise to stick to placing the CR 15 [mm] inferiorly to the CoC, as this already partly mimics the physiological movement, or even beyond, towards 28 [mm], if the patient’s anatomy allows this.

## Figures and Tables

**Table 1:** All the calculated centre of rotation (CR) positions per subject and per laterality. The delta-Y and delta-X columns show the distance from the patient’s specific centre of condyle (CoC) point, which lies in (0, 0). A negative delta-Y value indicates a shift inferiorly of the CoC and a negative delta-X value indicates a shift in the posterior direction.

			Right CR		Left CR	
Patient	Sex	Age	Δ Y	Δ X	Δ Y	Δ X
1	M	19	−39.7	−5.7	−39.4	−9.2
2	M	18	−13.7	−3.4	−14.9	1.9
3	F	53	−13.5	−11.8	−14.7	−10.1
4	F	20	−15.0	−0.8	−25.0	7.5
5	F	19	−33.6	−3.8	−21.7	−6.8
6	F	32	−26.6	−1.3	−28.0	−5.1
7	M	18	−45.9	−22.8	−39.4	−22.9
8	M	25	−3.2	−4.0	−24.5	−2.1
9	F	23	−23.7	−16.7	−24.7	−17.3
10	M	26	−29.7	−8.7	−31.9	−4.8
11	M	18	−23.0	−16.3	−33.7	−14.2
12	F	26	−19.9	1.0	−15.3	4.5
13	F	18	−33.8	1.2	−26.2	−1.6
14	M	46	−41.6	1.2	−43.5	1.2
15	M	47	−27.2	−13.6	−26.0	−8.5
16	F	47	−37.5	4.4	−26.9	2.1
17	M	23	−36.1	−1.7	−32.8	−1.9
18	M	44	−21.9	1.8	−23.7	0.6
19	M	40	−44.2	−1.2	−42.8	−6.7
20	M	29	−36.7	−11.7	−17.5	−11.2
		Mean	−28.3	−5.7	−27.6	−5.2

## Data Availability

The authors declare that the data supporting the findings of this study are available within the paper.
